# Supplementary material for: Prevalence and incidence of possible vascular dementia among Mexican older adults: Analysis of the Mexican Health and Aging Study
Source: PLoS One. 2021 Jul 8;16(7):e0253856. doi: 10.1371/journal.pone.0253856 (PMC8266048; doi:10.1371/journal.pone.0253856)
Supplement: S1 Table — provides general characteristics of individuals with incident possible vascular dementia comparing those who reported history of stroke in 2012 and 2015. Results show no significant differences in sociodemographic, cardiovascular conditions, depressive symptoms (≥5), and global cognition among groups. (PDF) [file pone.0253856.s001.pdf]

S1 Table. General characteristics of incident possible vascular dementia by time of stroke register

Characteristics	Incident Possible Vascular dementia		p-value
	Stroke in 2012<br><br>n=31	Stroke in 2015<br><br>n=44	
Age, Mean (SD)	76.4 (11.1)	74.5 (11.3)	0.465
Sex (female)	20 (64.5)	23 (52.3)	0.291
Education, years (SD)	3.2 (4.2)	3.3 (4.6)	0.924
Hypertension	23 (74.2)	30 (68.2)	0.573
Diabetes	12 (38.7)	13 (29.5)	0.407
Heart attack	7 (22.6)	6 (13.6)	0.314
Depressive symptoms*	23 (74.2)	30 (68.2)	0.573
Global cognition	-1.16 (0.8)	-1.21 (0.85)	0.808

P-value from t-test for continuous variables and Chi-square for categorical variables. Values in parentheses are weighted percentages derived using the MHAS sampling weights. MHAS= Mexican Health and Aging Study. *Depressive symptoms are presented for direct respondents only (2012=13096; 2015=11162), proxy respondents did not complete the depressive symptoms scale based on the study questionnaire.
