# Supplementary material for: Prevalence and incidence of possible vascular dementia among Mexican older adults: Analysis of the Mexican Health and Aging Study
Source: PLoS One. 2021 Jul 8;16(7):e0253856. doi: 10.1371/journal.pone.0253856 (PMC8266048; doi:10.1371/journal.pone.0253856)
Supplement: S2 Table — shows the results of the full logistic regression models for each source of attrition compared to responders. Being male, older age, having history of diabetes and heart attack and lower global cognition were predictors of mortality. Attrition due to lost was associated with lower age, higher education, living in urban areas, having history of stroke, lower hypertension, and lower global cognition. Refusals were predicted by lower age, higher education, and lower rates of hypertension. (PDF) [file pone.0253856.s002.pdf]

S2 Table. Logistic regression models predicting attrition at follow-up

	Decedents			Lost			Refusal		
	OR	p-value	95% CI	OR	p-value	95% CI	OR	p-value	95% CI
Males	1.49	0.000	[1.28, 1.73]	1.09	0.318	[0.92-1.29]	0.97	0.833	[0.77, 1.23]
Baseline Age	1.08	0.000	[1.07, 1.09]	0.97	0.000	[0.96, 0.98]	0.97	0.000	[0.95, 0.98]
Years of education	1.01	0.392	[0.98, 1.03]	1.09	0.000	[1.07, 1.11]	1.09	0.000	[1.06, 1.12]
Residence (urban)	1.10	0.230	[0.97, 1.33]	2.00	0.000	[1.58, 2.54]	2.43	0.000	[1.70, 3.47]
Baseline Stroke	1.39	0.103	[0.93, 2.07]	2.28	0.002	[1.34, 3.87]	NA	NA	NA
Baseline Diabetes	2.10	0.000	[1.78, 2.43]	0.90	0.355	[0.72, 1.12]	0.78	0.134	[0.57, 1.07]
Baseline Heart Attack	2.18	0.000	[1.63, 2.90]	1.09	0.726	[0.66, 1.78]	0.80	0.605	[0.35, 1.83]
Baseline Hypertension	1.14	0.097	[0.97, 1.33]	0.75	0.003	[0.62, 0.94]	0.75	0.024	[0.58, 0.96]
Baseline Global Cognition	0.64	0.000	[0.57, 0.71]	0.84	0.002	[0.75, 0.94]	0.98	0.818	[0.84, 1.14]
