# Supplementary material for: Prevalence and incidence of possible vascular dementia among Mexican older adults: Analysis of the Mexican Health and Aging Study
Source: PLoS One. 2021 Jul 8;16(7):e0253856. doi: 10.1371/journal.pone.0253856 (PMC8266048; doi:10.1371/journal.pone.0253856)
Supplement: Attachment — Submitted filename: Response to reviewers.docx [file pone.0253856.s003.docx]

April 8, 2021

Claudia K. Suemoto
Academic Editor
PLOS ONE Journal

We would like to thank the editor for his helpful suggestions. We also thank the reviewers for their generous comments on the manuscript. We have addressed all the suggestions in our current version which we allow ourselves to review and edit with the direction of your comments.

In this letter, **our responses are written in purple**. We have included a marked-up copy (pdf) of the manuscript where changes are highlighted in yellow.

**In response to Journal’s additional requirements:**

1. **Please review your reference list to ensure that it is complete and correct.**

- Thank you for identifying this. We have corrected 2 references [22 and 28] that had a broken e-link.

2. **Please ensure that your manuscript meets PLOS ONE's style requirements, including those for file naming.**

- Thank you for identifying the problem. We have corrected the Title page to accommodate PLOS ONE, Title, Autor, Affiliations formatting guidelines. We included a full and short title (page 1, lines 1-3) and used symbols to indicate author contributions and included the corresponding author’s e-mail address (page 1, line 14 and 15).
- We added an author, José A. Ávila Funes (page 1, line 5), who was not included in the initial submission. We acknowledge our error but want to correct it, as this author meets criteria for authorship, and must be included.
- We removed the key word section in the abstract (originally on page 3, line 51 and 52).
- We also corrected figure citations according to guidelines (page 6, line 114 and 121).

3. **We note that you have included the phrase “data not shown” in your manuscript**.

- We have removed this sentence originally located on pg. 11, lines 228-230 of the unrevised manuscript. The data was not a core part of the research being presented in our study.

4. **In your Data Availability statement, you have not specified where the minimal data set underlying the results described in your manuscript can be found.**

- We have added, in the manuscript’s methodology section (page 6, lines 109 and 110) as well as in the revised cover letter, an e-link to access the MHAS data and documentation, which are of public use and available at [*www.mhasweb.org*](http://www.mhasweb.org)*.* If specific data is required, please let us know, so we can share the requested information.

**In response to Reviewer #1 comments:**

5. **Introduction**: **(…the authors should bring information about dementia and vascular dementia mortality rates and burden to the health system (costs, for example).**

- Thank you for your recommendation. To increase the relevance of recognizing vascular cognitive impairment we have updated the first paragraph with information on dementia’s mortality and cost of care (page 4, lines 61-66).

6. **Still in the introduction, pg 11, line 81, I would quote that “a meta-analysis reported rates of post-stroke dementia that ranged from 7.4% […] to 41.3% […]”.**

- We have made this correction, page 4, line 79.

7. **Methods: When reading the section “Cognitive assessment”, I suggest the authors to cite the reference for the criterion of overall dementia for self-respondents (pg 7, line 134).**

- We appreciate that you have found this. We have added the missing reference (page 7, line 142).

8. **Methods: The effect of gender on IADLs for the Latin population (some men do not usually prepare a meal and some women may not manage money) – was that considered?**

**-** We are very thankful that you have noticed this. We have added a comment about the effect on gender in the Mexican population on page 7, lines 145-147).

9. **Methods: Was it possible to separate IADL disability due to cognitive impairment and not due to stroke complications?**

- We appreciate your comment. We have included this issue on the limitations section of the study (page 20, lines 380-382).

10. **Methods: (In pg 5, line 101) – is this the ethical approval? This information needs to be more specific.**

- As correctly noted in page **6 line 106**, the Institutions mentioned are responsible for ethical approval of the MHAS protocol.

11. **Figures, tables, and results.  I suggest that, throughout the text, the results can be cited with only one decimal place. “**At age 75 years and older individuals had 3.6 greater odds of incident PVD…” and line 295: “Being in the lowest quartile of the education distribution increased in 2.8 the odds of incident PVD”.

- We are thankful for your suggestion. Decimals on page 16, line 296 and 297, have been modified, as well as all decimals in figures, tables, and results throughout the manuscript.
- For decimals with minor numeric intervals (e.g., 0.48 – 0.50) we chose to include the exact 2 decimal digit at the corresponding table’s footnote (page 14, 272, 273) and at one citation (page 4, line 78).

12. **Conclusions**. **The authors should mention which differences there are that could explain this variability, with references (e.g., use of different PVD criteria and cognition scales?).**

- We have made this distinction by explaining, in the Introduction (page 5, lines 84-93) section of the Manuscript, how methodological differences between studies, such as the use of different diagnostic criteria, the inclusion of additional cognitive evaluation tests, and studies’ neuroimaging availability, could account for variability in results.

13. **Conclusions.** **In the limitation section of the discussion, the fact two thirds of the PVD population were diagnosed by proxy respondents may have made the criterion for detecting PVD less sensitive, so the authors should mention that.**

- We have included a statement regarding this issue on page 20, lines 388-390.

14. **Conclusions. Second, the exclusion of new cases of dementia without history of stroke is also a limitation and must be explained through the manuscript, such as patients with cerebral small vessel disease without typical stroke history and evolving with dementia.**

- We have learned a lot from your comments and are deeply grateful for your them**.** We have added a statement about this issue in the limitations section (page 20, lines 383-386) of our study.

15. **I suggest the authors make a careful analysis of grammatical and spelling errors, as: Pg 17, line 316 – “found an increasing pattern”, Pg 18, line 332: “hypothesis where pre-existing mechanisms” and Pg 18, line 340: methodological.**

- We are very thankful for noticing this. A careful grammatical and spelling analysis has been made and errors have been corrected (page 18, line 330 and 340).

**In response to Reviewer #2 comments:**

16. **Were the weights of MHAS that are needed to have prevalence and incidence rates representative of the Mexican population used**?

- The 2001 baseline survey of the MHAS includes a nationally representative sample of individuals born prior to 1951, that is the population aged 50 or older as of 2001. The sample for the MHAS baseline was selected from residents of both rural and urban areas and distributed in all 32 states of the country in urban and rural areas. A new sample of adults born between 1952-1962 was added in 2012. This information is explained elsewhere, and we added the reference for this on page 6 line 104 and 105.

17. **In the Discussion, the authors need to discuss how using a criterion attached to the presence of stroke influence the prevalence and incidence of vascular dementia. They need to make clearer that the findings probably underestimate the rates of VaD in this study.**

- We agree and are thankful for your comments. We believe we have addressed this issue in the Discussion section (page 19, lines 346-349) of our study, as well as in page 20 lines 382-390.

We believe the manuscript is now suitable por publication in PLOS ONE.

Thank you for your consideration.

Sincerely.

Corresponding Author
Sara G. Aguilar Navarro

On behalf of all authors.
