# Supplementary material for: Prevalence and incidence of possible vascular dementia among Mexican older adults: Analysis of the Mexican Health and Aging Study
Source: PLoS One. 2021 Jul 8;16(7):e0253856. doi: 10.1371/journal.pone.0253856 (PMC8266048; doi:10.1371/journal.pone.0253856)
Supplement: Attachment — Submitted filename: Response to reviewers2.docx [file pone.0253856.s004.docx]

June 8, 2021

Claudia K. Suemoto
Academic Editor
PLOS ONE Journal

We would like to thank academic editor for her helpful suggestions. We also thank the reviewers for their generous comments on the manuscript. We have addressed all the suggestions in our current version which we reviewed and edited accordingly.

In this letter, **our responses are written in purple**. We have included a marked-up copy of the manuscript where changes are highlighted in yellow.

**In response to Journal’s additional requirements:**

1. **Please review your reference list to ensure that it is complete and correct.**

- Thank you for identifying this. We have corrected a misplaced reference (#17 instead of #15) in page 5, line 94.
- We also noted a misplaced reference on page 6 line 110-111. The web address where information can be downloaded is [www.mhas.org](http://www.mhas.org). The aim and methodological design of the MHAS is indeed published elsewhere, and reference 19 (<https://doi.org/10.1093/ije/dyu263>) is correctly placed at page 6 line 106.
- We added a previously misplaced reference (#45) at page 21 line 399 (previously referenced as #40)
- We also removed any bold formatting we used for references.

**In response to reviewer #2:**

1. In my previous review, I have requested information on the use of sampling weights. However, the authors did not answer this comment. They provided information about the sample procedure. In epidemiologic studies, we cannot evaluate the whole population, so we sample some individuals. To make the sample representative of the population, we can weight the analyses by the sampling weights. This is a common procedure in studies that aim to measure prevalence and incidence rates. Please, provide information if you used the weights. If you have not used them, recalculate your analyses.

- We are very thankful of your comment. Yes, we used MHAS sampling weights. We now added a sentence at the statistical analysis section (page 10, line 192-193) including the reference to the paper that explains the MHAS sampling weights. We also added this information to each of the tables where weighted data is presented; Table 1 (page 13, line 244-245), Table 2 (page 15, line 274-275), Table 3 (page 16, lines 292-293), and Supporting information table 1 (S1_table).

We believe the manuscript is now suitable por publication in PLOS ONE.

Thank you for your consideration.

Sincerely.

Corresponding Author
Sara G. Aguilar Navarro

On behalf of all authors.
